# Genome-wide association studies of photosynthetic and agronomic traits in cowpea collection

**DOI:** 10.1093/g3journal/jkae233

**Published:** 2024-10-04

**Authors:** Olakunle Sansa, Michael Terrance Abberton, Johnson Ariyo, Rajneesh Paliwal, Adenike Ige, Ibnou Dieng, Moninuola Ayo-Vaughan, Victor Idowu Olowe, Olaniyi Oyatomi

**Affiliations:** International Institute of Tropical Agriculture, IITA, Ibadan 200132, Nigeria; Department of Plant Breeding and Seed Technology, Federal University of Agriculture, Abeokuta 110124, Nigeria; International Institute of Tropical Agriculture, IITA, Ibadan 200132, Nigeria; Department of Plant Breeding and Seed Technology, Federal University of Agriculture, Abeokuta 110124, Nigeria; International Institute of Tropical Agriculture, IITA, Ibadan 200132, Nigeria; Department of Agronomy and Plant Genetics, University of Minnesota, St. Paul, MN 55108, USA; International Institute of Tropical Agriculture, IITA, Ibadan 200132, Nigeria; Department of Plant Breeding and Seed Technology, Federal University of Agriculture, Abeokuta 110124, Nigeria; Department of Plant Breeding and Seed Technology, Federal University of Agriculture, Abeokuta 110124, Nigeria; International Institute of Tropical Agriculture, IITA, Ibadan 200132, Nigeria

**Keywords:** SNP markers, drought tolerance, photosynthesis, marker-assisted selection, cowpea, Plant Genetics and Genomics

## Abstract

Exploring genomic regions linked with drought tolerance and photosynthesis in cowpea could accelerate breeding of climate-resilient cowpea varieties. A genome-wide association study (GWAS) was conducted to identify marker–trait associations for agronomic and photosynthetic traits measured under well-watered and water-stressed conditions. One hundred and twelve cowpea accessions from IITA were phenotyped for agronomic and photosynthetic traits across 3 locations in 2 years: Ibadan, Ikenne (2020 and 2021), and Kano (2021 and 2022). The accessions were genotyped using 19,000 Diversity Arrays Technology sequencing single-nucleotide polymorphism (SNP) markers from which 9,210 markers were utilized for GWAS analysis using BLINK and linear mixed model (LMM) in GAPIT. Results revealed significant accession × environment interactions for measured traits, while ΦPSII, ΦNO, and ΦNPQ had significant and consistent correlations with grain yield across conditions. GWAS identified 5 SNP markers having consistent associations with grain yield under well-watered and water-stressed conditions and 3 markers associated with ΦNPQ and ΦNO. Gene annotations revealed *Vigun04g169000* and *Vigun08g168900* genes linked with grain yield and highly expressed under water-stressed conditions have functional roles in regulating plant development and adaptive response to environmental stress. *Vigun07g133400*, *Vigun07g132700*, and *Vigun07g258000* genes linked with ΦNPQ and ΦNO are involved in activities controlling photoprotection and stress-induced damage in plants. This study identified natural genetic variation in cowpea and correlations between photosynthetic traits and grain yield under real-field drought conditions. The identified SNP markers upon validation would be valuable in marker-assisted selection and useful for cowpea breeders to harness the role of photosynthesis in genetic enhancement of cowpea’s tolerance to drought.

## Introduction

Cowpea [*Vigna unguiculata* (L.) Walp] is an important grain legume that constitutes a valuable source of protein in the diets of millions of people in Africa. It is predominantly grown by smallholder farmers in the dry agro-ecological regions of sub-Saharan Africa and is widely cultivated in Asia and America (Dadson *et al*. 2005; Singh *et al*. 2014; Boukar *et al*. 2019). Cowpea production is constrained by many biotic and abiotic factors. Drought is one of the major challenges affecting cowpea production (Hall 2004; Muchero *et al*. 2009; Boukar *et al*. 2016). Fatokun *et al*. (2012) reported the ability of cowpea to tolerate drought and thrive under low-fertility soils; however, the response of cowpea genotypes to drought differs significantly. Drought affects different stages of development in cowpea and is more critical during the reproductive phase, which leads to reduced yield or total crop loss. Due to climate change, the frequency and intensity of drought coupled with heat stress pose significant threats to cowpea production and sustainable food security in Africa (Hall *et al*. 2003). Drought negatively affects essential processes like photosynthesis, transpiration, and stomatal conductance, which results in the alteration of, assimilates partitioning, plant metabolic activities, decreased leaf area, reduction in plant height, and number of leaves per plant (Fahad *et al*. 2017; Merwad *et al*. 2018). Photosynthesis plays a crucial role in determining crop yield and any detrimental effect on this process significantly affects the overall productivity of the plant. Additionally, photosynthesis is important for crop biomass improvement, primarily by maximizing light interception and optimizing radiation use efficiency, as indicated by Furbank *et al*. (2015), Ort *et al.* (2015), Singh *et al*. (2014), and Furbank *et al*. (2020). Therefore, enhancing plant productivity requires understanding photosynthetic activities under optimum and stress conditions (Singh and Reddy 2011).

The increasing threat of drought to cowpea production underscores the critical need for development of drought-tolerant cowpea cultivars. According to Mickelbart *et al*. (2015) and Valliyodan et al. (2017), the genetic diversity present in germplasm may possess alleles that are adapted to extreme environments; harnessing and utilizing this diversity may lead to the improvements of drought-tolerant cultivars. The development of several strategies to promote germplasm utilization by plant breeders has allowed breeders to effectively harness the diverse and valuable genetic potential available in Gene Banks for crop improvement. These strategies include core collection (Frankel 1984; Brown 1989), minicore collection (Upadhyaya and Ortiz 2001), and Focused Identification of Germplasm Strategy (FIGS) (Mackay *et al*. 2005). The Genetic Resources Center of the International Institute of Tropical Agriculture (IITA) holds large cowpea germplasm which can provide useful alleles for climate-resilient traits including drought tolerance (Fatokun *et al*. 2012). Exploring these untapped alleles in diverse genetic resources of IITA could potentially help to understand the genetics of drought tolerance and reveal valuable traits that contribute to enhanced resilience in cowpea plants under drought conditions.

The emergence of next-generation sequencing (NGS) technologies has offered a powerful approach to exploring genetic diversity and uncovering new markers (Korte and Farlow 2013). While progress has been made through conventional breeding in cowpea, the availability of new molecular genetic tools enables the application of modern breeding strategies for cowpea improvement (Gupta et al. 2015). Diversity Arrays Technology (DArT) has experienced an increasing interest worldwide because it has efficiently satisfied the requirements of throughput, genome coverage, and highly informative single-nucleotide polymorphism (SNP) markers (Jaccoud *et al*. 2001). The advances in genomic technologies have also enabled a better understanding of the genetic basis of variation using genome-wide association studies (GWAS), as it can be used for identification and high-resolution mapping of useful genetic variability from germplasm sets that have resulted from many rounds of historical recombination (Yu and Buckler 2006). GWAS uses a high-resolution method to identify genes or genomic regions that are associated with a trait of interest. The identification of functional genes and alleles associated with specific traits through GWAS has significant implications for crop improvement and breeding programs, as it enables the targeted selection and manipulation of desired traits to enhance crop performance (Xue *et al*. 2013; Liu and Yan 2019).

The application of marker technology has significantly accelerated the progress in developing novel genetic and genomic resources for cowpea breeding. This advancement has increased the use of molecular markers in cowpea improvement programs. Boukar *et al*. (2019) and Chamarthi *et al*. (2019) have highlighted the progress made on cowpea with the use of genomic resources.

GWAS have been reported to be a powerful approach for identifying causal genes linked with complex traits like drought, yield, and photosynthesis which have provided an understanding of the genetic basis of these traits. The uncovering of causal genes enables the deployment of modern breeding strategies to develop improved crop varieties. For example, Zafar *et al*. (2024) identified genes controlling root system architecture and stress response in cowpea which holds value in developing drought-tolerant cowpea varieties. Paudel *et al*. (2021) also identified candidate genes linked with flowering time in cowpea which provides a pathway for breeding early maturing and adaptable varieties in a changing climate. Other GWAS on cowpea have been conducted for various traits including pod length (Xu *et al.* 2017), black seed coat color (Herniter *et al*. 2018), seed size (Lo *et al*. 2019), drought and salt stress tolerance (Ravelombola *et al*. 2021), yield-related traits (Nkomo *et al*. 2022), and aphid resistance (Ongom *et al*. 2022). Furthermore, GWAS approach was also used to explore the genetic architecture of some photosynthesis traits in crops like cowpea, maize, soybean and rice. (Herritt *et al*. 2016; Wang *et al*. 2015, 2017; Wang and Yang 2020; Wang and Hsub 2020; Wu *et al*. 2021; Wei *et al*. 2022; Yi *et al*. 2023). The use of GWAS to identify marker–trait associations (MTAs) has also played a vital role in accelerating breeding programs as functional validations, and testing of these SNP markers in large populations and diverse genetic backgrounds aids marker-assisted selection allowing breeders to select desirable lines at the early phase of their breeding programs (Wu *et al*. 2021; Ige et al. 2022; Codija *et al*. 2022; Potts *et al*. 2024).

The limited progress in increasing drought tolerance in cowpea can be attributed to the polygenic nature of drought and the insufficient understanding of the underlying genetic mechanism of cowpea response to drought. Genome-wide association mapping offers an opportunity to identify MTAs and understand the genetic architecture of complex traits providing a better understanding of plant response under drought stress conditions. The complex nature of photosynthesis has been a major challenge in linking photosynthetic efficient phenotypes with yield under real-field conditions and stress environments. Our study provides strong directions for improving photosynthesis by exploring natural diversity in cowpea to identify possible links between photosynthetic traits and yield under real-field drought conditions as well as genomic regions driving drought tolerance and photosynthesis which is an important step toward trait selection and marker-assisted breeding for climate-resilient cowpea varieties with improved yield. Therefore, the objectives of this research were to (i) evaluate the genetic variability for yield-related and photosynthetic traits in some core collections of cowpeas assessed under well-watered and water-stressed conditions and (ii) identify SNP markers associated with yield-related and photosynthetic traits under well-watered and water-stressed conditions.

## Materials and methods

### Genetic materials

Cowpea accessions were obtained from the Genetic Resources Center of IITA, and 50 accessions each from the FIGS and minicore subset were selected based on the same origin of collection representing countries majorly growing cowpea across sub-Saharan Africa. In addition, 2 standard checks, drought-tolerant TVu-17360 (Dan Ila) and drought-susceptible TVu-7778), and 10 reported drought-tolerant genotypes by Agbicodo *et al*. (2009) and Fatokun *et al*. (2012) give a total of 112 accessions used in the study (Supplementary Tables 1–3).

### Field evaluation

Field experiments were conducted in 3 research stations of IITA located in Nigeria: Ibadan, Oyo State (7° 38ʹN, 3° 89ʹ E); Ikenne, Ogun State (6° 86ʹN, 3°71ʹ E); and Minjibri, Kano State (12° 00ʹN, 8°31ʹ E). Field experiments were conducted during the dry seasons at the 3 locations of the study (Ibadan, November–February 2020/2021 and 2021/2022; Ikenne, November–February 2020/2021 and 2021/2022; and Kano, October–January 2021/2022 and February–May 2022). Ibadan is a derived savannah, while Ikenne is a humid forest, although the research location at Ikenne falls between derived savannah and humid forest. Minjibri, Kano, is a Sudan savannah region that experiences a long and dry season with rains between July and September. The experiments were arranged in 2 different water regimes (well-watered and water-stressed) and laid in a 8 × 14 alpha lattice design with 3 replications. Cowpea seeds were planted at 2 seeds per hill on a 1-m single-row plot with a spacing of 0.20 m within rows and 0.75 m between rows. The well-watered and water-stressed plots were separated by 20-m spacing to prevent water drift during drought imposition. The 2 plots were subjected to irrigation twice a week. Irrigation was supplied to the well-watered plots from the day of planting until harvesting, while the water-stressed plots received irrigation for only 35 days after planting, after which drought was imposed until plant maturity initiating reproductive stage drought stress. Aside from the different irrigation treatments, weed control was done through manual weeding, and insect pests were controlled when necessary.

### Data collection

Agronomic data were collected from the well-watered and water-stressed plots as described in Supplementary Table 4. Photosynthesis data were collected on the water-stressed plots across all locations using a MultispeQ device version 2.0 developed by PhotosynQ Inc., Michigan, USA. To measure photosynthesis, 1 plant from each plot was chosen at random, and its uppermost leaf part was tagged. Two sets of measurements were taken on the tagged leaf, 1 was taken before stress imposition, and the other was taken when the entire water-stressed plot displayed significant signs of drought stress. The photosynthesis measurements were observed in non-cloudy and low-windy days when the sun was completely visible and measurements were done per accession in each block within a replicate. All accessions within each replicate were captured in a day with subsequent replicates captured on consecutive days. Measurement time per sampling ranged from 20 to 35 s with the day and time of capturing factored in as covariates in the data analysis. During sampling, we ensured that the photosynthetic active radiation (PAR) sensor on the MultispeQ device faces the direction of sunlight. In addition, cowpea leaves are broad in shape, and they completely covered the light guide of the MultispeQ device. The photosynthesis parameters observed and their description are presented in Table 1, while Table 2 shows the means of humidity, temperature, and PAR captured during photosynthesis measurements.

**Table 1.** Description of photosynthesis traits measured by the MultispeQ device developed by PhotosynQ.

Parameters	Description
Ambient humidity (AH)	The amount of moisture in the air in a particular environment expressed in percentage (%)
Ambient temperature (AT)	The average temperature of an environment expressed in degrees Celsius (°C)
Photosynthetic active radiation (PAR)	The portion of the light spectrum utilized by plants for photosynthesis (µmol m⁻² s⁻¹)
Relative chlorophyll content (RCC)	Concentration of chlorophyll in the leaf is used as an indicator of plant nitrogen content and indicator of stress in plants
Leaf angle	The inclination between the midrib of the leaf blade and the vertical stem of a plant
Leaf temperature differential (LTD)	The difference between leaf temperature and ambient temperature in degrees Celsius
Linear electron flow (LEF)	A proximate measurement of photosynthesis that measures how much light is being moved around in the chloroplast following exposure to light
NPQt (non-photochemical quenching)	Measures how much of the incoming light is being dissipated as heat
ΦPSII	Quantum yield of photosystem II measures the percentage of incoming light (excited electrons) that goes into the photosynthetic process where most light energy is converted into food
ΦNO	Measures the ratio of incoming light lost via nonregulated process. Combination of a number of unregulated processes whose by-products can inhibit photosynthesis or be harmful to the plant.
ΦNPQ	Measures ratio of incoming light that goes toward non-photochemical quenching. Plant regulating excess energy to reduce damage to the plants

**Table 2.** Mean of relative humidity, ambient temperature, and photosynthetic active radiation captured during measurement of photosynthesis traits.

Condition	Location	Year	Humidity (%)	Temperature (°C)	PAR (µmol m^−2^ s^−1^)
Before stress imposition	Ibadan	2020/2021	41.61	37.85	1183.60
During stress imposition			36.78	38.33	1196.59
Before stress imposition	Ibadan	2021/2022	31.76	35.39	1096.40
During stress imposition			23.76	36.91	1148.01
Before stress imposition	Ikenne	2020/2021	46.76	36.26	1147.51
During stress imposition					
Before stress imposition	Ikenne	2021/2022	48.81	35.84	983.56
During stress imposition			41.73	37.67	1050.05
Before stress imposition	Kano	2020/2021	29.47	32.58	1574.42
During stress imposition					
Before stress imposition	Kano	2021/2022	22.49	33.35	1405.01
During stress imposition			17.41	30.85	1462.51

### Data analysis

Analysis of variance (ANOVA) was conducted using the Statistical Analysis System (SAS Institute, 2012) software, and a general linear model procedure (PROC GLM) was adopted. Before analysis, data quality checks were performed, and outliers were removed. In addition, data were confirmed to follow normal and independent distributions validating model assumptions for ANOVA. A combined ANOVA was done separately on all the data collected in well-watered and water-stressed plots and for photosynthesis traits measured before and during stress imposition. Each combination of water regime and location within a year was treated as a distinct environment, resulting in 6 environments each for each water regime as described in Supplementary Table 5. In the ANOVA, a RANDOM statement with the TEST option was employed. The random factors include environments, replication within environments, and blocks (nested within the replication × environment interaction), while accessions were treated as a fixed factor. A single best linear unbiased estimate (BLUE) for the agronomic and photosynthetic data across all test environments for each water regime, broad-sense heritability, and phenotypic correlations were generated using the linear mixed model (LMM) in META-R as described by Alvarado *et al.* (2020) as shown below. For agronomic traits, the model is described in equation 1 while equation 2 describes the model for photosynthetic traits with date and time of measurement fitted as covariates.


(1) $$\begin{array}{r} Y_{ijkl}=\mu +Env_{i}+Rep_{j}+Block_{k}(Env_{i}\;Rep_{i})+Gen_{i}+Env_{i}\;\times \;Gen_{i}+\varepsilon_{ijkl} \end{array}$$

(2) $$Y_{ijkl}=\mu +Env_{i}+{\mathrm{Rep}}_{j}+Block_{k}(Env_{i}+{\mathrm{Rep}}_{i})+Gen_{i}+Env_{i}\times Gen_{i}+Cov+\varepsilon_{ijkl}$$

where $Y_{ijkl}$ is the trait of interest, μ is the mean effect, $Env_{i}$ and $Gen_{i}+Env_{i}$ are the effects of the *i*th environment and the environment by genotype interaction, $Rep_{j}$ is the effect of the *i*th replicate, $Block_{k}(Env_{i}\;Rep_{i})$ is the effect of the *k*th block within the *i*th replicate and *i*th environment, *Cov* is the effect of the covariate, and $\varepsilon_{ijkl}$ is the error associated with the *i*th environment, *j*th replication, and *k*th block.


$$Broad\;Sense\;Heritability\;(H^{2})=\;\frac{\sigma_{g}^{2}}{\sigma_{g}^{2}+\frac{\sigma_{ge}^{2}}{\mathrm{nEnvs}}+\;\frac{\sigma_{\varepsilon }^{2}}{\left(nEnvs\times nRep\right)}}$$

where *σ*^2^g, *σ*g^2^e, and *σ*^2^*ε* are the genotypes, G × E is the interaction and error variance components, *n*Rep is the number of replicates, and *n*Envs is the number of environments.


$$\mathrm{Phenotypic}\;\mathrm{correlation}\;pg_{ij}=\frac{\;pg_{ij}}{h_{i}h_{j}}$$

where ρ *p i j* is the phenotypic correlation between environments *i* and *j* and ℎ*i* and ℎ*j* are the square roots of heritability of environments *i* and *j*, respectively.

### Genome-wide association studies (GWAS) for drought tolerance and photosynthesis in cowpea

#### Genotyping and quality control

One hundred accessions consisting of FIGS and minicore subset were genotyped for this experiment. The extraction protocol for DArT sequencing (DArT-Seq) was employed for extracting genomic DNA from collecting leaf samples from 3-week-old cowpea seedlings. DNA quality checks were assessed using 1% agarose gel electrophoresis, while a NanoDrop 2000 spectrophotometer (Thermo Scientific, Waltham, MA, USA) was used to quantify the extracted DNA. Following the protocols of Jaccoud *et al*. (2001), a concentration of 100 ng/μL high-quality DNA was sent for genotyping to DArT Pty Ltd, in Australia (https://www.diversityarrays.com). The DArT-Seq whole-genome profiling methods used for complexity reduction, cloning, library construction, and cleaning were described by Egea *et al*. (2017). A total of 19,000 DArT-Seq SNPs were generated from 100 accessions of cowpea population high-depth DArT-Seq SNP genotyping. The physical position of all discovered SNPs on cowpea population was determined by aligning SNPTags on the cowpea reference genome *Vigna unguiculata* v1.1 of elite African variety IT97K-499-35 (https://phytozome-next.jgi.doe.gov/info/Vunguiculata_v1_1. (Lonardi *et al*. 2019). All these 19,000 SNPs were subjected to quality control checks for removing poor-quality SNPs. Furthermore, a call rate of ≥ 70%, average reproducibility ≥ 95%, missing data < 0.20, and minor allele frequency (MAF) ≥ 0.01 were used as criteria for filtering out poor-quality SNPs. In addition, 2 accessions (TVu-10005 and TVu-12432) were filtered out due to low-quality SNPs. After filtering, 9,210 SNP markers were utilized for population structure and GWAS analysis.

### Association analysis

The Trait Analysis by Association, Evolution and Linkage (TASSEL) v.5.2 software was used to generate the principal component matrix (P) and kinship matrix (K). The PCA (P) which includes the first 5 principal components and kinship matrix (K) derived from all the markers were fitted as covariate variables to reduce the false positives due to population stratification and control spurious association (Bradbury *et al*. 2007; Yu and Buckler 2006). GWAS analysis was conducted using GAPIT (Genetic Association and Prediction and Integrated Tools)—R package (Lipka *et al*. 2012). BLUE values of the agronomic and photosynthetic traits across all test environments were utilized for GWAS analysis in both well-watered and water-stressed environments. The best-fitted model for GWAS was determined based on the quantile–quantile (QQ) plot (Okunlola *et al.* 2023). In our study, a LMM was best fitted for agronomic traits, while Bayesian-information and Linkage-disequilibrium Iteratively Nested Keyway (BLINK) model was best fitted for photosynthetic traits. BLINK provides a Bayesian framework for robust inference under complex genetic architectures with the ability to identify true-positive signals more efficiently for low-heritability traits as obtained in our study for photosynthesis traits and other studies (Huang *et al*. 2019; Cebecci et al. 2023).

The Bonferroni correction −log10 (*P*) > 5.46 (*P* = 0.05/*N*; *N* = total markers used) had a more stringent threshold, and when tested, it produced few significant MTAs. Hence, we used the false discovery rate (FDR) approach which has been reported to be more powerful in controlling the proportion of false positives (type I errors) while detecting true positives (Benjamini and Hochberg 1995; Verhoeven et al. 2005; Ongom et al. 2022). FDR method was applied in R using the *p.adjust()* function and setting the method to “fdr” which adjusts the GWAS *P-values* according to Benjamini and Hochberg procedure. An average FDR threshold was then calculated from the adjusted *P-values* at 5% significance level as shown below:


$$FDR=(\alpha \times 100)/\left(\overset{1}{\underset{i}{\sum }}\;p.adjust\right)$$

where *FDR* = is the false discovery rate threshold and *α* refers to the acceptable level of type I error which was set to 0.05 in this study. $\sum_{i}^{1}\;p.adjust$ is the sum of adjusted *P-values* for each SNP extracted from the R output. $\sum_{i}^{1}\;p.adjust\;$ from this study = 9132.07. Hence, a GWAS threshold of −log10 ^(FDR)^ was used to declare significant MTAs as described below:


$$FDR=[(0.05\times 100/9132.07)=5.47\times 10^{-4},\mathrm{thus}\text{–}log10^{\left(FDR\right)}=3.3$$

The Manhattan plot generated by the cM plot function in R was used to visualize the distribution of SNPs across the entire cowpea chromosome. Linkage disequilibrium (LD) was calculated for SNP pairs across different distances using plink v1.9 (Chang et al. 2015) while the LD decay curve was plotted using the ggplot2 function in R. Following the identification of significant MTAs, the positions of SNP markers having consistent significant MTA across conditions of the study were mapped on cowpea genome v.1.1 using the JBrowse genome browser to discover candidate genes proximal to the location of each SNP on the genome (Ongom *et al*. 2022). The functions of the candidate genes identified were assessed through the European Molecular Biology Laboratory (EMBL) and Universal Protein Resource (UniProt) databases.

## Results

### Phenotypic variations

**Fig. 1.**
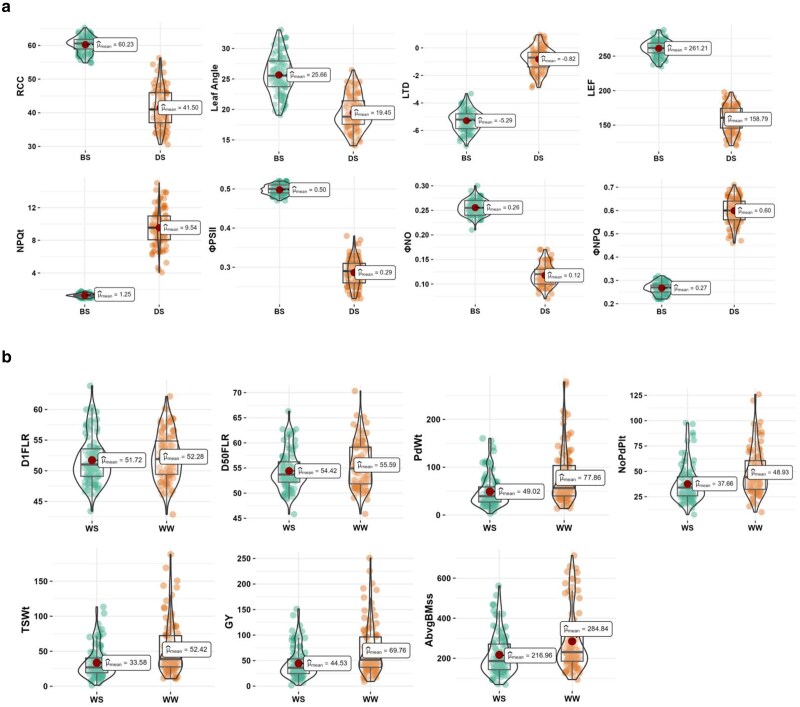
a and b) Violin plots showing the distribution and means from BLUEs of all cowpea accessions measured for photosynthetic and agronomic traits across test conditions. BS, before stress; DS, during stress; WW, eell-watered; WS, water-stressed.

The mean square values from combined ANOVA and broad-sense heritability estimates for agronomic and photosynthetic traits across test conditions are presented in Table 3. Significant accession × environment interactions were found for all agronomic traits measured across well-watered and water-stressed conditions. However, for photosynthesis traits measured before stress imposition, significant accession × environment interactions were only found for relative chlorophyll content, NPQt, and ΦPSII. During stress conditions, all photosynthetic traits except leaf angle and NPQt had significant accession × environment interaction. Broad-sense heritability estimates for agronomic traits under well-watered conditions ranged from 51% for days to first flowering to 86% for pod weight, while under water-stressed conditions, it ranged from 64% for days to 50% flowering to 80% for pod weight. Broad-sense heritability estimates of photosynthesis traits measured before and during stress imposition ranged from 1% for leaf angle to 61% for LTD and 1% for LEF to 50% for RCC, respectively. The comprehensive ANOVA tables are presented in Supplementary Tables 6–8. Violin plots showing the distribution and means from BLUEs of all cowpea accessions measured for photosynthetic and agronomic traits across test conditions are displayed in Fig. 1a and b. Mean values for all agronomic traits measured were higher under well-watered conditions when compared to water-stressed conditions. Mean values for photosynthetic traits measured before stress conditions were higher except for leaf temperature differential, NPQt, and ΦNPQ having higher mean values during stress conditions.

**Table 3.** Mean squares and broad-sense heritability estimates from combined ANOVA of agronomic and photosynthetic traits for 112 cowpea accessions.

Agronomic				
	Well-watered		Water-stressed	
	Accession × ENV	H^2^ (%)	Accession × ENV	H^2^ (%)
Days to first flower	98.42**	53	76.53**	65
Days to 50% flowering	141.47**	51	77.59**	64
Pod weight (g)	6156.14**	86	2917.75**	80
Number of pods per plant	1873.98**	72	1221.32**	73
Total seed weight (g)	2830.39**	85	1424.04**	78
Grain yield (g/m^2^)	5033.15**	85	2509.24**	79
Aboveground biomass (g)	82691**	80	59731**	64
Photosynthesis				

	Before stress		During stress	
	Accession × ENV	H^2^ (%)	Accession × ENV	H^2^ (%)
RCC	52.21**	45	224.39**	50
Leaf angle	84.469	1	71.165	8
LTD	2.14	61	5.57**	-
LEF	1150.65	6	4415.18**	1
NPQt	0.65**	24	49.47	27
ΦPSII	0.001*	55	0.013**	23
ΦNO	0.002	43	0.004**	28
ΦNPQ	0.004	57	0.028**	26

ENV, environment.

* and ** are significant at 0.05 and 0.01 probability levels, respectively.

### Correlations between grain yield and photosynthetic traits

**Fig. 2.**
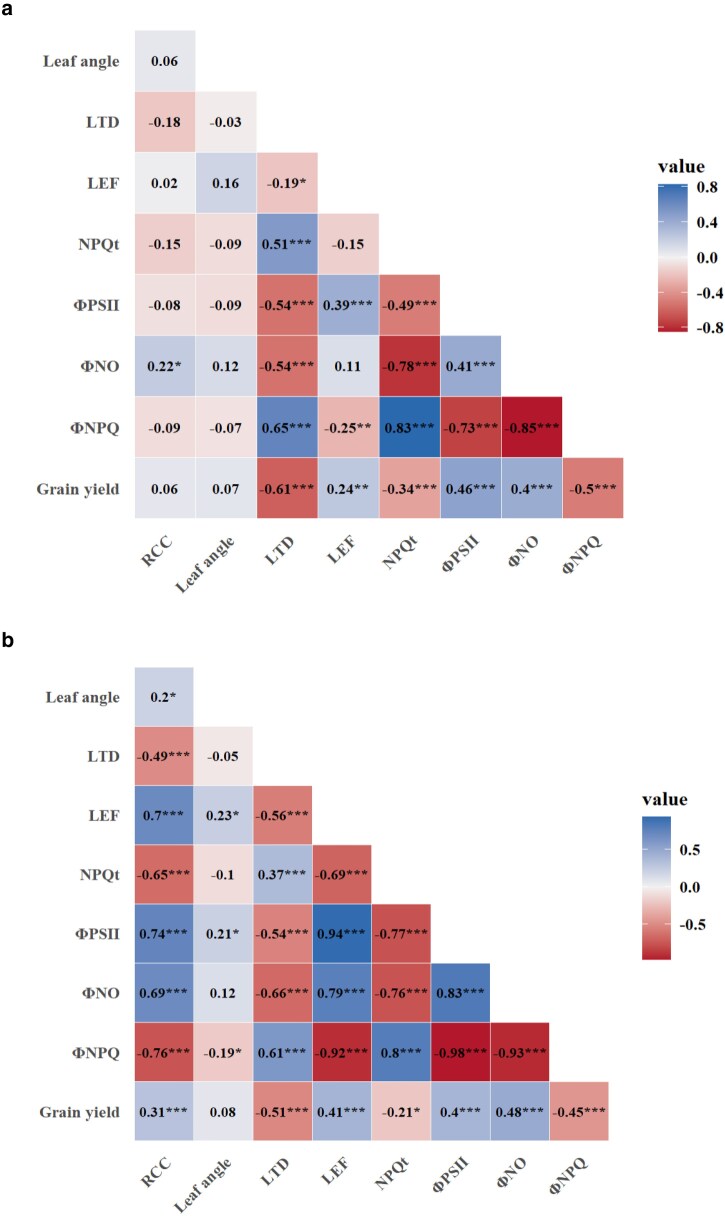
a and b) Correlation coefficients between grain yield and photosynthetic traits under a) non-stress and b) stress conditions.

Correlation coefficients between grain yield and photosynthetic traits are presented in Fig. 2a and b. Grain yield and relative chlorophyll content (RCC) exhibited significant and positive correlations under stress conditions, while no correlation was recorded under non-stress conditions. Significant and negative correlations were observed between grain yield and leaf temperature differential (LTD) under non-stress and stress conditions. Correlation between grain yield and linear electron flow (LEF) was significant and positive at both stress and non-stress conditions, while significant and negative correlations were observed between NPQt and grain yield. Significant and positive correlations were found between grain yield and ΦPSII under stress and non-stress conditions. Similar significant and positive correlations were found between ΦNO and grain yield. Conversely, grain yield and ΦNPQ showed significant and negative correlations under stress and non-stress conditions.

### GWAS of agronomic and photosynthetic traits

**Fig. 3.**
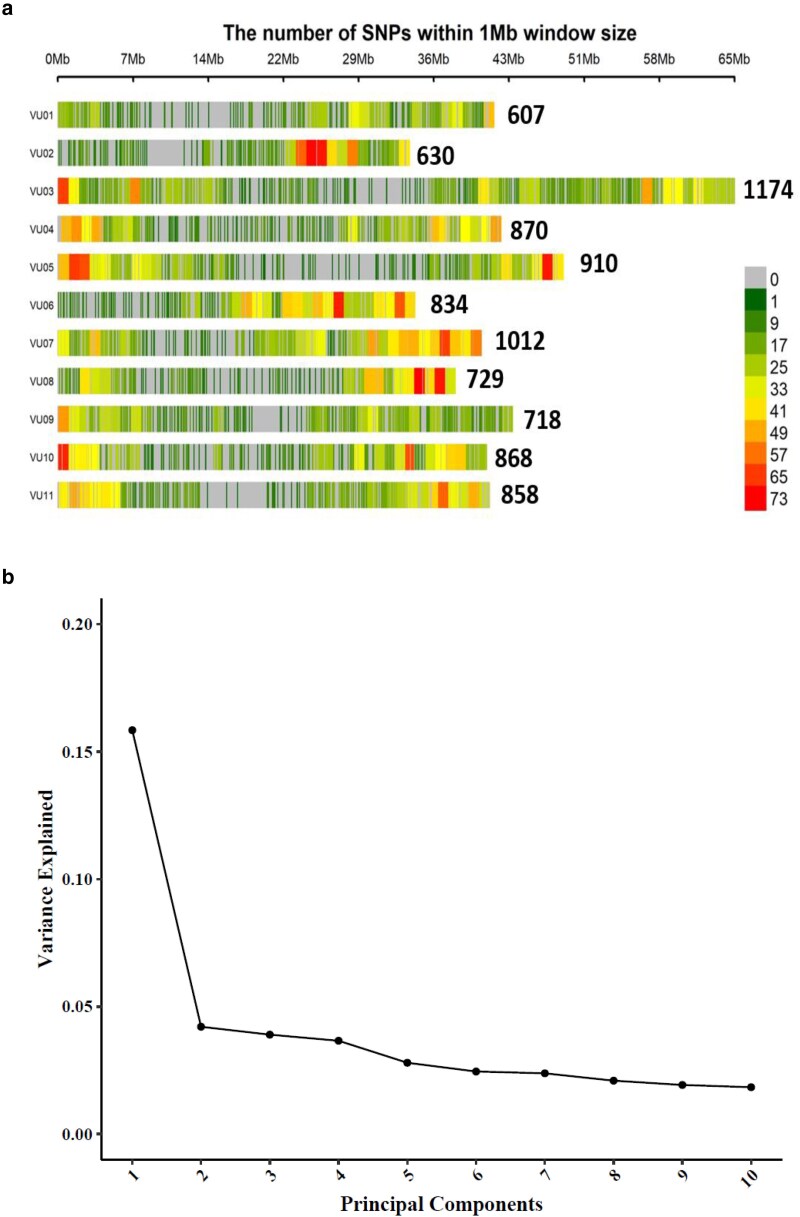

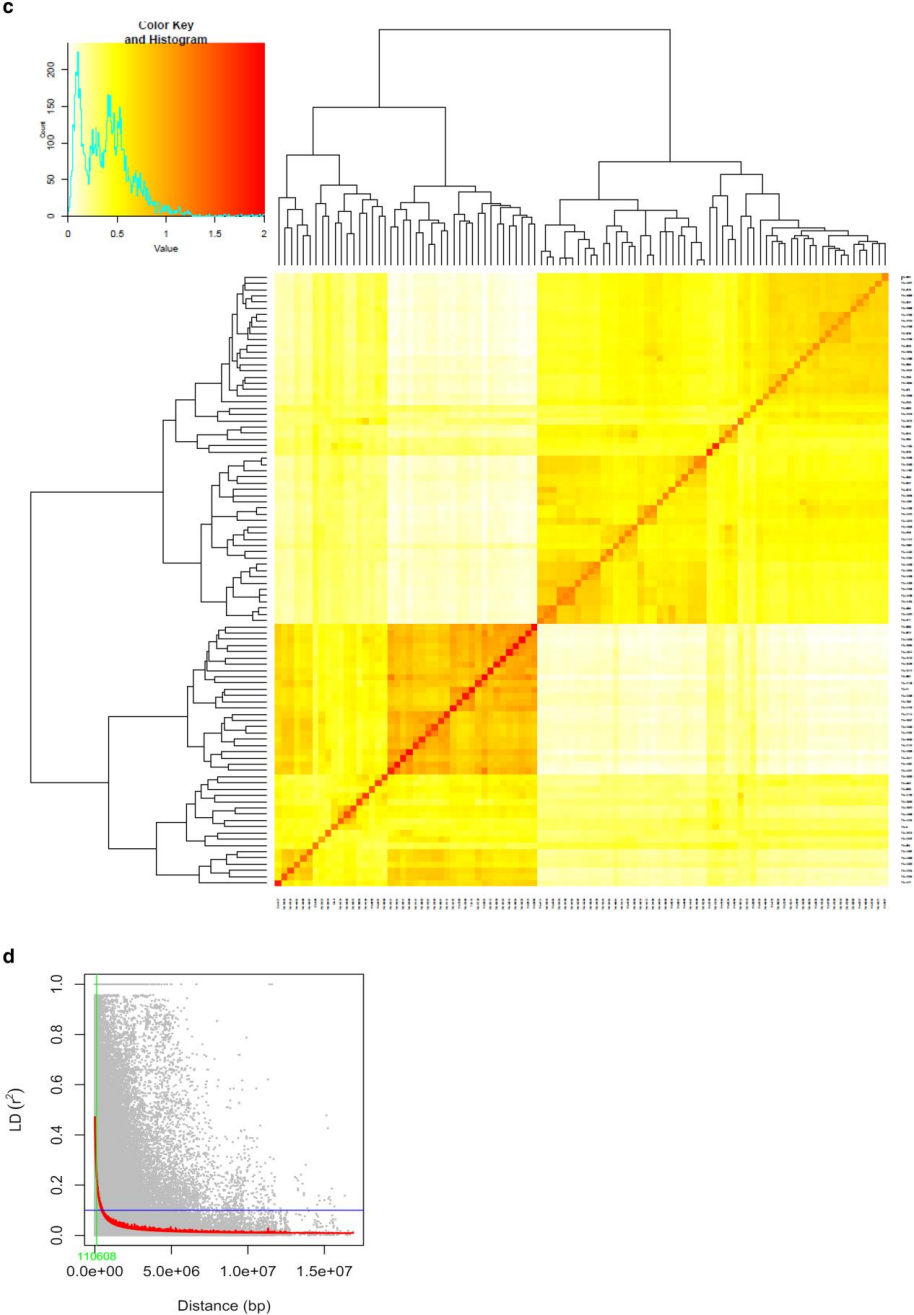
a) Distribution of SNP markers across 11 chromosomes of cowpea. b) PCA and percentage variance for 9210 DArT-Seq markers. c) Heat map showing the results of kinship matrix. d) LD *r*^2^ plotted against physical distance (bp) for the 98 cowpea accessions.

**Fig. 4.**
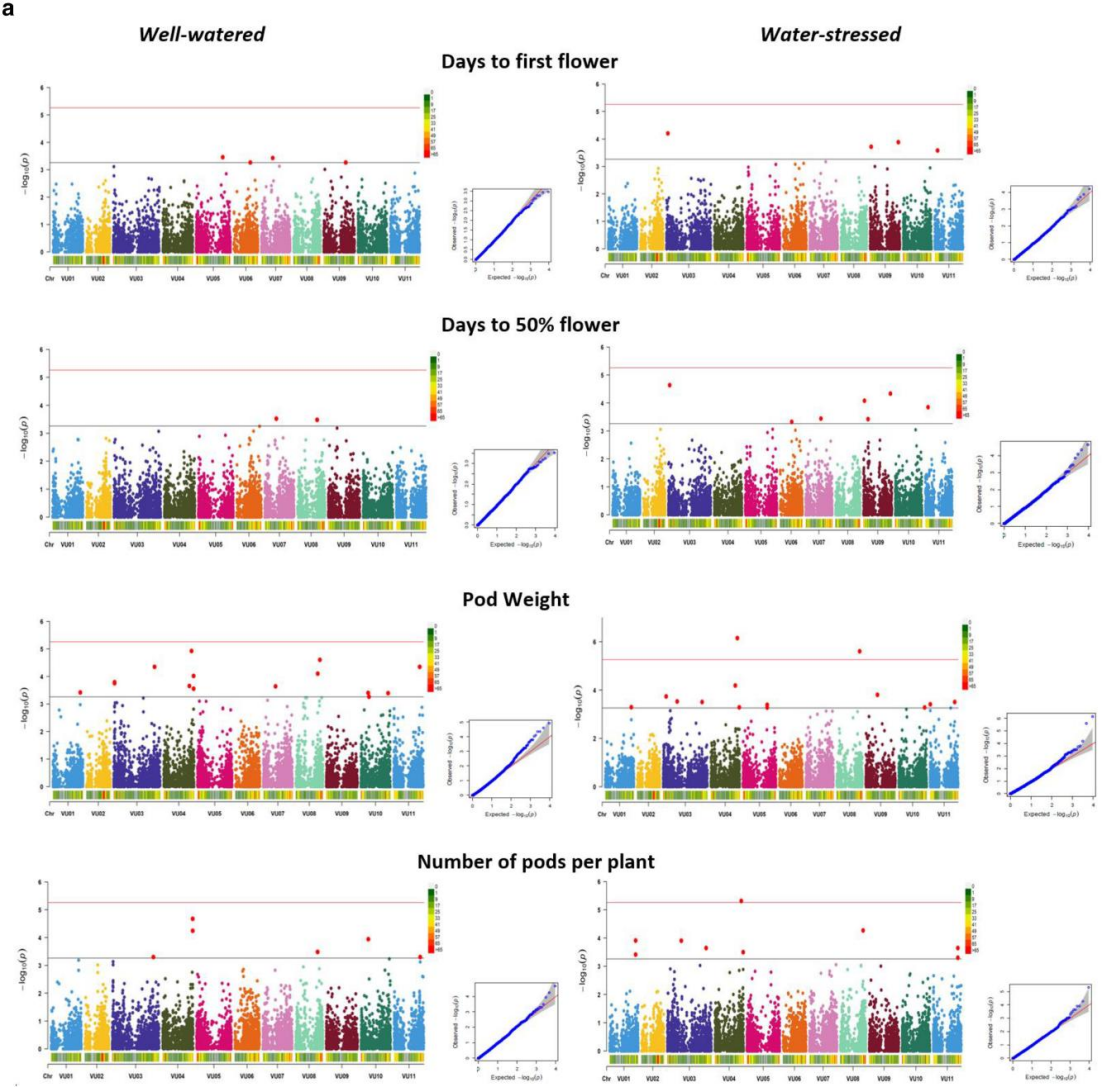

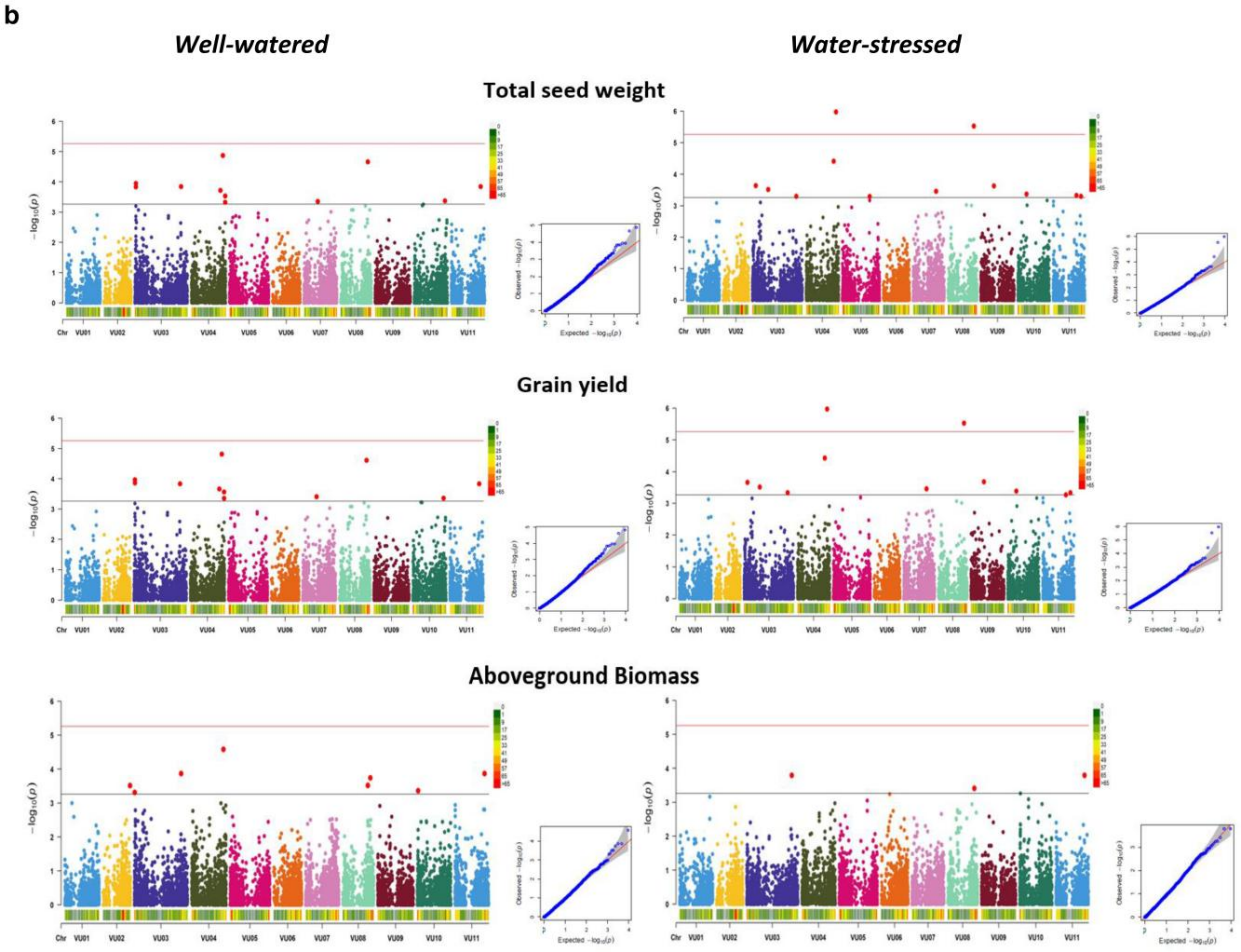
a and b) Manhattan and Q + Q plots showing SNPs associated with agronomic traits under well-watered and water-stressed conditions.

**Fig. 5.**
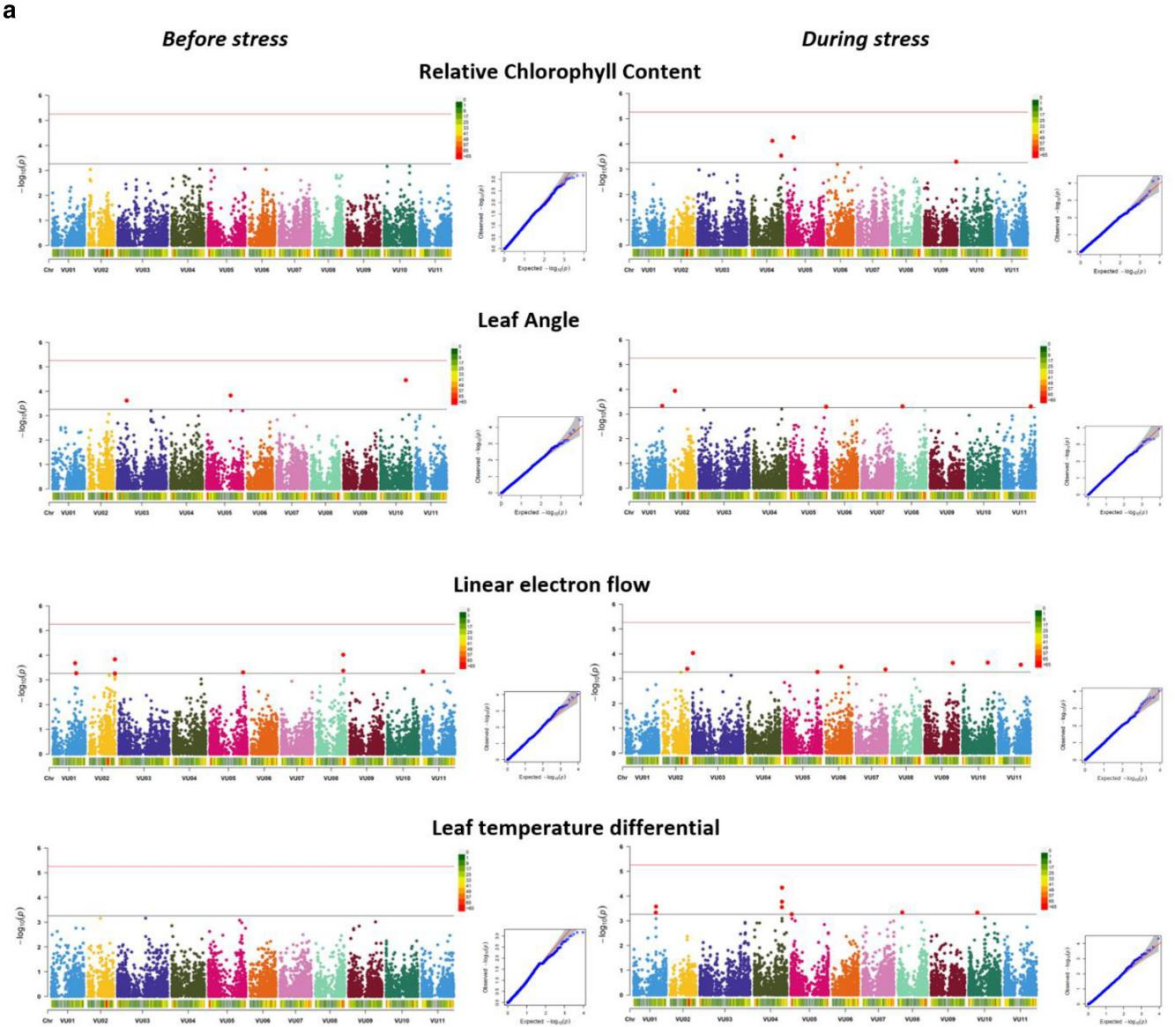

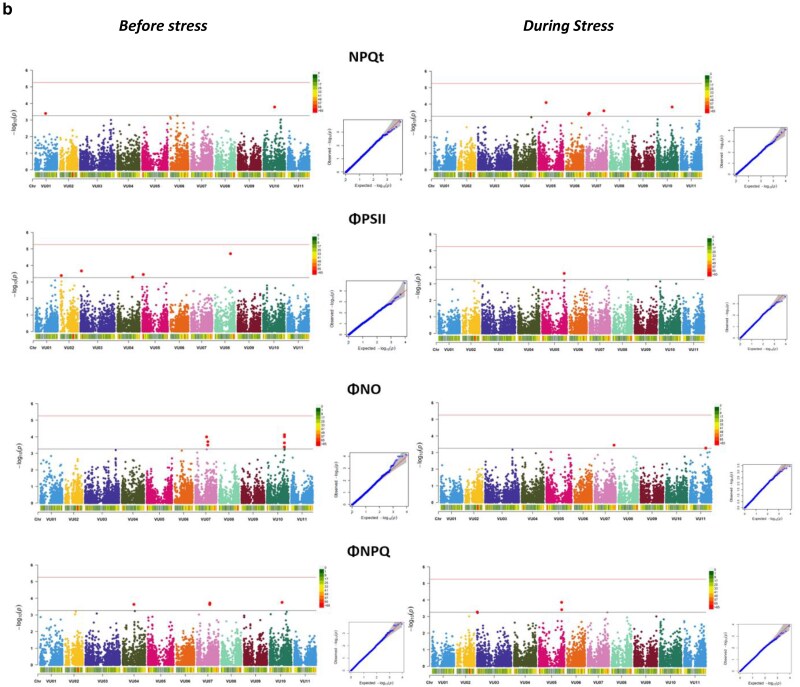
Manhattan and Q + Q plots showing SNPs associated with photosynthetic traits measured before and during stress imposition.

The distribution of 9,210 SNPs across the 11 chromosomes of cowpea is shown in Fig. 3a. SNP coverage ranged from 609 for chromosome 1 to 1174 for chromosome 3, and the average marker resolution across the genome was calculated to be 51,945.39 base pairs (≈52 Kb). Figure 3b shows a scree plot for principal component axis (PCA) and the variance contribution of each axis. The kinship matrix showing the genetic relationships within the population is presented in Fig. 3c. The 5 PCAs used in the GWAS analysis accounted for 30.41% of the total genetic variation. The LD maximum (*r*^2^) value was 0.43 and was found to decay to 0.1 at <11 Kb (Fig. 3d). Table 4 shows the summary of significant MTAs for all measured traits. Under well-watered conditions, 57 significant MTAs were identified, while a total of 53 markers were found under water-stressed conditions with pod weight having the highest number of significant MTAs under both conditions. For photosynthetic traits measured before stress imposition, 33 significant MTAs were found for all traits except for relative chlorophyll content and leaf temperature differential, while during stress imposition, 40 significant markers were identified. Figures 4 and 5 show the Manhattan and QQ plots showing SNP marker distribution across the chromosome as well as the significant MTAs for agronomic and photosynthetic traits. All the identified SNP markers and their *P-values* are presented in Supplementary Tables 9–12. SNP markers having consistent associations with more than 1 trait and across stress conditions are presented in Table 5. Markers Vu04_39340965 and Vu08_34011809 showed high significance above the Bonferroni threshold under water-stressed conditions and were consistently associated with pod weight, number of pods per plant, total seed weight, and grain yield. However, when FDR significant test was applied, markers Vu04_39340965, Vu08_34011908, Vu03_57303579, and Vu11_36490988 had consistent associations with pod weight, number of pods per plant total seed weight, grain yield, and aboveground biomass under well-watered and water-stressed conditions. Furthermore, markers Vu04_38317304 and Vu04_36182035 had consistent associations with pod weight and grain yield, respectively, under well-watered and water-stressed conditions. Three markers on chromosome 7 had consistent associations with 2 photosynthetic traits (ΦNPQ and ΦNO) with 2 of the markers (Vu07_24318383 and Vu07_24248530) identified before stress and marker Vu_37527811 identified during stress condition.

**Table 4.** Summary of SNP markers associated with agronomic and photosynthetic traits measured in the study.

Agronomic traits								
Condition	D1FLR	D50FLR	PDWT	NoPdPlt	TSWT	GY	AbvgBmss	
Well-watered	2	2	15	6	12	12	8	
Water-stressed	4	7	11	9	9	10	3	

Photosynthesis traits								
	RCC	Leaf angle	LTD	LEF	NPQt	ΦPSII	ΦNO	ΦNPQ
Before stress	-	3	-	8	3	5	5	9
During stress	4	5	9	9	5	1	3	4

D1FLR, days to first flower; D50FLR, days to 50% flowering; PdWt, pod weight; NoPdPlt, number of pods per plant; TSWt, total seed weight; GY, grain yield; AbvgBMss, aboveground biomass.

**Table 5.** SNP markers with consistent associations and their corresponding candidate genes across test conditions.

Marker	Chr_Position	Traits	Conditions	Gene ID	Physical position on genome	Encoding protein
14074811|F|0-12:G > A-12:G > A	Vu04_39340965	PDWT, TSWT, GY	WW and WS	Vigun04g169000	Vu04: 39340084..39343374 forward	Plastid movement impaired1
14055805|F|0-23:C > G-23:C > G	Vu08_34011908	PDWT, TSWT, GY, AbvgBmss	WW and WS	Vigun08g168900	Vu08: 4003617..34004379 reverse	GRP family
14056089|F|0-34:G > A-34:G > A	Vu03_57303579	PDWT, NoPdPlt TSWT, GY AbvgBmss	WW and WS	Vigun03g370600	Vu02: 57306186..57308087 reverse	CBL-interacting serine/threonine-protein kinase 12-related
14057278|F|0-16:C > T-16:C > T	Vu11_36490988	PDWT, NoPdPlt TSWT, GY, AbvgBmss	WW and WS	Vigun11g155600	Vu11: 36487600..36491555 forward	Protein kinase apk1a, chloroplastic-related
14057194|F|0-42:A > T-42:A > T	Vu04_38317304	Pod weight	WW and WS	Vigun04g161000	Vu04: 38311716..38312980 forward	Oxidoreductase, 2OG-FE II oxygenase family protein
4075093|F|0-47:G > A-47:G > A	Vu04_36182035	Grain yield	WW and WS	Vigun04g146500	Vu04: 36178790..36182162 forward	Ancient conserved domain protein-related
42215292|F|0-60:T > A-60:T > A	Vu07_24318383	ΦNPQ, ΦNO	Before stress	Vigun07g133400	Vu07:24316079..24320574 forward	Organic solute transporter-related
14075587|F|0-24:G > A-24:G > A	Vu07_24248530	ΦNPQ, ΦNO	Before stress	Vigun07g132700	Vu07:24247840..24249044 forward	Germin-like protein subfamily 2 member 1-related
25364932|F|0-32:C > A-32:C > A	Vu07_37527811	ΦNPQ, ΦNO	During stress	Vigun07g258000	Vu07:37527711..37529163 reverse	UDP-glycosyltransferase 84a1-related

Chr, chromosome; PDWT, pod weight; TSWT, total seed weight; GY, grain yield; AbvgBmss, aboveground biomass.

## Discussion

### Phenotypic variation for agronomic traits

The performance of the 112 cowpea accessions used in this study varied significantly under well-watered and water-stressed conditions indicating that the accessions were of diverse genetic backgrounds. This provides an opportunity to select accessions that can thrive under both optimum and drought stress conditions. Watanabe *et al*. (1997) and Fatokun *et al*. (2012) screened cowpea germplasm and breeding lines for drought stress tolerance; they found variation in the cowpea lines evaluated and identified germplasm lines with better tolerance to drought than many of the breeding lines and varieties used in their study. Fatokun *et al*. (2012) further suggested that utilizing grain yield performance under drought conditions could serve as a criterion for the selection of cowpea lines exhibiting enhanced levels of drought tolerance.

### Phenotypic variation for photosynthetic traits

Enhancing photosynthesis is considered a promising strategy for improving the yield of crops, as highlighted in studies by Kromdijk *et al.* (2016), Wu *et al*. (2019), and Souza *et al*. (2022); however, available genetic variation for photosynthesis traits in crop germplasm resources would be required (Fernández-Calleja *et al*. 2020). The study showed the existence of significant genotypic variation in the photosynthetic traits observed before and during stress imposition. The significant variation in the study environments shows that the environments were unique, and the cowpea accessions responded differently before and during stress imposition. The interaction between accession and environment was also evident in some traits. The variations in photosynthetic performance among the accessions studied can be attributed to the impact of light, temperature, and water use efficiency (Fernández-Calleja *et al*. 2020). Fahad *et al*. (2017) and Merwad *et al*. (2018) reported that drought causes a negative impact on the essential photosynthesis process which leads to the alteration of the plant metabolic activities and assimilate partition. Similar trends were observed in this study, the imposition of drought stress led to a decrease in the chlorophyll content, LEF, ΦPSII, and ΦNO. Gnankambary *et al*. (2020) noted that water deficit leads to a significant loss in chlorophyll, decrease in photosynthetic activity, and reduction in seed yield production. The decrease in chlorophyll under stress led to an increase in the NPQt process. This corroborates with the findings of Basu *et al*. (2016) suggesting that such an increase in NPQt signifies the detachment of light-harvesting complexes from photosynthetic reaction centers. This adaptive response is considered a mechanism to mitigate drought-induced damage to photosynthesis. Murchie and Lawson (2013) reported the use of chlorophyll fluorescence to monitor the photosynthesis performance of plants. Maxwell and Johnson (2000), Sarkar *et al*. (2009), and Kalaji *et al*. (2016) also noted the importance of chlorophyll in the early detection of response to stress and the ability of the plant to tolerate environmental stresses. Genotypic variation in chlorophyll content observed during stress therefore provides an opportunity to select cowpea accession that maintains high chlorophyll content under stress conditions, and this can be a pathway to identifying drought-tolerant accessions from the cowpea germplasm resources. The increase in leaf temperature observed during stress suggests potential stomatal closure which is a mechanism for plants to preserve their water status. High leaf temperature results in a decrease in transpiration rate and has a strong link to stomatal closure. However, measuring stomatal conductance validates the linkage between leaf temperature and stomatal closure (Fernández-Calleja *et al*. 2020). There was a decrease in linear electron flow under stress imposition. Rott *et al*. (2011) reported the decrease in LEF is derived from a decrease in carbon dioxide (CO_2_) assimilation. Leaf angle plays a crucial role in photosynthesis as it can be impacted by light interception, transpiration, and competition among plants. The orientation and positioning of leaves determine how efficiently they capture sunlight, affecting the overall photosynthetic process (Anten 2005; Nilsen and Forseth 2018). Findings from this study revealed significant variation in leaf angle, and there was also a decrease in leaf angle during stress conditions when compared to before stress conditions. These responses observed in leaf angle may be attributed to variations in light, heat, and drought as suggested by Yang *et al*. (2023). Moreover, Pearcy *et al*. (2005) highlighted that a change in orientation or angle of incidence results in the difference in intercepted radiation by 22% which has an impact on photosynthesis, leaf temperature transpiration, and energy balance. In addition, biological and environmental factors also affect leaf angle which results in variation within the same environment and among the same individual as reported by Yang *et al*. (2023), while similar results were observed in this study, the angle of the MultispeQ may also have contributed to variations observed. In this study, NPQt increased during stress imposition, and the implication of this is that it plays a critical role in how plants respond to stress as it constitutes a major mechanism for avoidance of photodamage and a significant avenue in the dissipation of excess energy. Fernández-Calleja *et al*. (2020) found similar results for high NPQt in barley hybrids. These findings were also supported by the report of Brestic *et al*. (1995) who noted that the dissipation of excess excitation energy at the level of the ΦPSII antennae has been proven to be the major protective mechanism against the deleterious effects of high light in dehydrating leaves. In this study, it was observed that a high ΦPSII led to a reduction in ΦNO, but a significant increase was observed for ΦNPQ and NPQt. Similar findings were reported by Ben-Jabeur *et al*. (2022). When plants experience drought, one of the mechanisms to save water is stomata closure, and this initiates a decrease in CO_2_ concentration which in turn leads to excess energy (Huang *et al.* 2012). However, if this excess energy is not safely dissipated, it becomes harmful to ΦPSII due to overreduction of reaction centers and the increased production of photooxidative reactive oxygen species (ROS) in the chloroplast (Loggini *et al*. 1999). To ensure plant conversion of light energy into food, it is important to monitor and protect ΦPSII, and according to Huner *et al*. (1996), Wu and Bao (2011), Huang *et al*. (2012), and Zingaretti *et al*. (2013), plants initiate different mechanisms to protect ΦPSII which include activation of ΦNPQ, reduction of ΦPSII efficiency, and activation of antioxidative pathways. Fernández-Calleja *et al*. (2020) further proposed exploring the role and mechanisms of ΦNPQ and ΦNO as potential parameters for indirect selection and breeding for water stress tolerance.

### Broad-sense heritability of agronomic and photosynthetic traits

To incorporate a trait of interest into a breeding program, it is important to consider the heritability of such traits. Breeders employ heritability as a metric to gain insights into the degree to which traits are inherited by the progenies in subsequent generations (Piepho and Möhring 2007). The observation of moderate-to-high broad-sense heritability in grain yield and other related traits measured in this study implies that these traits are likely to be dependable for the direct selection of cowpea genotypes with drought tolerance. Belko *et al*. (2014) noted that cowpea grain yield under optimum conditions showed higher heritability when compared to grain yield under stress conditions. Similar results were also observed in this study with grain yield heritability higher under well-watered conditions when compared to water-stressed conditions suggesting the impacts of environmental factors on yield potential of crops when subjected to stress. Furthermore, high heritability indicates genetic factors account for a large proportion of observed variations in yield performance, while low heritability indicates environmental factors or genotype by environment interactions contribute to most of the observed variations. Higher heritability estimates offer effective and reliable selection by plant breeders. Adewale *et al*. (2020) also reported that high heritability in yield-related traits increased the efficiency of GWAS and the true identification between an SNP marker and a candidate gene.

Low-to-moderate heritability was observed in most of the photosynthetic traits. Several studies have also reported low heritability in photosynthetic traits (Pelleschi *et al*. 2006; Wang *et al*. 2013; Ziyomo and Bernardo 2013; Khodadadi *et al*. 2014; Čelp *et al*. 2016; Flood *et al*. 2016; Qu *et al*. 2017; Prado *et al*. 2018). For example, movement during measurement and changes in the angle of the MultispeQ may have contributed to the large influence of environmental factors leading to a low heritability estimate. This partly explains the limitation in the use of photosynthesis traits by plant breeders despite the valuable phenotypic variation and multiple years of selection aimed at improving crop productivity (Long 2014). However, we found reasonable heritability in our study, and this shows that when proper efforts and time are put in place to manage the influence of environmental conditions, good heritability estimates are achievable for photosynthesis even in diverse environments. Moreover, the advent of high-throughput phenotyping allows large-scale phenotyping in multiple environments in a shorter time, and this could help plant breeders and physiologists understand the plasticity of photosynthetic traits and their response to varying environmental conditions thereby providing insights and directions in improving the heritability of photosynthetic traits.

### Correlations between agronomic and photosynthetic traits

This study explored the correlation between photosynthetic and grain yield across non-stress and stress conditions. Consistent correlations were found between photosynthesis traits and grain yield except for relative chlorophyll content which showed correlations with grain yield only under stress conditions. LTD, NPQt, and ΦNPQ had negative correlations, while ΦPSII and ΦNO exhibited positive correlations with grain yield across test conditions. Thus, improving photosynthetic traits like LTD, NPQt, ΦNPQ, and ΦPSII may enhance photosynthetic activities and make plants more resilient to environmental stresses thereby potentially increasing overall yield. For example, cowpea accessions from this study with lower leaf temperature under stress suggest better water use efficiency and could serve as potential parents in breeding for drought-tolerant and drought-adaptable cultivars. In addition, selection of cowpea accessions with high ΦPSII and lower NPQt and ΦNPQ could be beneficial in breeding for climate resilience as these accessions may be efficient in utilizing light energy for increased photosynthesis leading to improved crop productivity. These findings provide direction for breeders on incorporating photosynthetic traits in selection indices to develop drought-tolerant and high yielding cowpea varieties and also provide insights for targeted genetic modifications of photosynthetic traits to improve yield in cowpea. Therefore, using MultispeQ to measure plant photosynthetic processes and their contribution to yield can enhance our understanding of plant performance, leading to more effective selection strategies for crop improvement.

Condon *et al*. (2004) and Silva-Pérez *et al*. (2020) reported that diurnal changes in the surrounding environment can lead to wrong estimation of the photosynthetic potential of crops which suggests that care should be taken when selecting crops with improved photosynthetic performance. These challenges in studying photosynthesis explain some of the limitations on the usage of photosynthesis traits by plant breeders. Fernández-Calleja *et al*. (2020) used two MultispeQ devices simultaneously to phenotype barley hybrids and found highly significant variation for traits measured in the unstressed and stressed plots. They further concluded that although the use of two MultispeQ devices increased the speed of operation, it however resulted in an additional source of experimental error. Sales *et al*. (2022) also reported the influence of environment on phenotypic variation in photosynthetic traits and noted the need for careful consideration when designing experiments to study photosynthesis under field and glasshouse conditions. Acevedo-Siaca *et al*. (2021) reported low heritability of photosynthesis traits in rice, furthermore, and no correlation between agronomic and photosynthetic traits. They concluded that photosynthesis remains unimproved despite the presence of significant phenotypic variations. Consequently, this may also explain the reason why improvements in photosynthesis have not translated into increased yields (Long 2014). Drawing insights from the findings and experience from this study, the challenges associated with fluctuating light intensity can be addressed using advanced high-throughput phenotyping such as the use of unmanned aerial vehicles (UAVs), drones which can increase the speed of measurement and can effectively phenotype large germplasm in a short time. In addition, when cost-effective and handheld high-throughput devices like MultispeQ are to be used, strong considerations should be given to the number of genotypes to be sampled. However, when more than one device is to be used, a good experimental design should be considered as this will be beneficial to capture any additional source of variation that may be introduced.

### GWAS of agronomic and photosynthetic traits

GWAS analysis identified 57 SNP markers associated with agronomic traits under well-watered and 53 markers under water-stressed conditions. Six of these markers were found to have stable associations with pod weight, number of pods per plant, total seed weight, grain yield, and aboveground biomass across well-watered and water-stressed conditions. However, two SNP markers on chromosomes 4 and 8 were found to be above the Bonferroni threshold under water-stressed conditions. This suggests that genes within the genomic region associated with these traits may have high expression levels under stress indicating their roles in stress response mechanisms. These markers could be useful in selecting cowpea genotypes with potential to tolerate drought and maintain good yield performance. Furthermore, this study identified 3 markers on chromosome 7 associated with ΦNPQ and ΦNO which suggests this genomic region might be involved in regulating photoprotection and energy dissipation thereby improving photosynthetic efficiency. In addition, these markers can be useful in screening and selection of cowpea genotypes with efficient use of light energy and higher photoprotective capacity under stress conditions. These findings further support the suggestions of Fernández-Calleja *et al*. (2020) who proposed exploring the role and mechanisms of ΦNPQ and ΦNO as potential parameters for indirect selection and breeding for water stress tolerance. Several studies have successfully identified quantitative trait loci (QTLs) that are significantly associated with complex traits such as drought tolerance and photosynthesis (Muchero *et al*. 2009; Burridge *et al*. 2017; Wu *et al*. 2021; Nkomo *et al*. 2022). The identified SNP markers hold potential in marker-assisted selection and could accelerate breeding for climate-resilient cowpea varieties. However, validation of these SNPs in line with diverse genetic backgrounds and across different test environments would be required before use in marker-assisted breeding programs for cowpea.

### Candidate genes for agronomic and photosynthetic traits

Markers Vu04_39340965 and Vu8_34011908 found to be highly expressed under water-stressed conditions harbored *Vigun04g169000* and *Vigun08g168900* genes encoding plastid movement impaired1 protein and glycine-rich protein (GRP) family, respectively. Plastid movement impaired1 protein is a plant-specific C2-domain protein required for efficient chloroplast photo-relocation movement in plant organelle. It has been found to play critical roles in light utilization for photosynthesis, fundamental cellular activities, and adaptive responses to environmental stress in plants (DeBlasio *et al*. 2005; Suetsugu *et al*. 2015). GRP family has been reported to be responsible for regulating plant development, plant defense, and control of stomata opening during osmotic stress (Ueki and Citovsky 2002, 2005; Yokoyama and Nishitani 2006; Park *et al*. 2007; Kim *et al*. 2008; Mangeon *et al*. 2010). In addition, *Vigun03g370600* and *Vigun11g155600* genes located within genomic regions of markers Vu03_57303579 and Vu11_36490988 had consistent associations with pod weight, number of pods per plant, total seed weight, grain yield, and aboveground biomass. These genes encode for CBL-interacting serine/threonine-protein kinase 12-related and protein kinase Apk1a, and chloroplastic-related proteins, respectively. CBL protein family has been reported to play important roles in plant and seed development and regulation of stress response (Kanwar *et al*. 2014; Ma *et al*. 2020; Verma *et al*. 2021; Poddar *et al*. 2022). Protein kinases are major regulatory components in almost all cellular processes and are critical for cellular signaling as they provide biochemical links between the perception of environmental stresses and the subsequent activation of cellular responses. Studies by Cheng *et al*. (2011), Wang and Yang (2020), and Wang and Hsub (2020) have also established the vital roles protein kinases play in the response of plants to stress like drought, high salinity, cold, and pathogen attack.

Furthermore, 2 markers on chromosome 7 were found to have consistent associations with ΦNPQ and ΦNO before stress imposition harboring *Vigun07g133400*, *Vigun07g132700*, and *Vigun07g258000* genes. *Vigun07g133400* encodes for organic solute transporter-related (OSTR) protein reported to be involved in regulating ion and solute transport and energy dissipation which is an important photosynthetic machinery in the thylakoid membrane crucial for NPQ activation. The presence of OSTR in regions associated with ΦNPQ and ΦNO suggests the associated marker is involved in regulating the photoprotective processes in the chloroplasts essential for optimizing photoprotection (Niyogi 1999; Spetea and Schoefs 2010). *Vigun07g258000* gene encodes for Germin-like proteins (GLPs) which are a group of proteins involved in wide range of functions in plants such as stress responses, cell wall synthesis, enzymatic reactions, and plant development. They possess superoxide dismutase (SOD) activity, which helps in the detoxification of ROS, a by-product of excessive light energy in photosynthesis. These findings suggest GLP2-1’s role in regulating ROS levels may potentially contribute to the efficient functioning of NPQ and reduction of energy loss through nonregulated dissipation pathways thereby enhancing photosynthetic efficiency (Bernier and Berna 2001; Dunwell et al. 2008; Sun *et al*. 2020). During stress condition, UDP-glycosyltransferase 84A1-related protein (UGT84A1) was found to be within genomic regions associated with ΦNPQ and ΦNO. UGT84A1 are a large family of enzymes involved in the glycosylation of various plant metabolites which activates plant hormones such as auxins or salicylic acid involved in stress responses. They are also involved in the glycosylation of flavonoids and other antioxidants, which play a role in mitigating oxidative stress by scavenging ROS (Bowles et al. 2006; Marrs, 1996). Glycosylation can modulate the function of these molecules, particularly in regulating protective responses to environmental stresses like drought, light intensity, and temperature indicating UGTs’ role in photosynthetic activities of ΦNPQ and ΦNO for improving photosynthesis and protection from stress-induced damage in plants (Chen *et al.* 2020; Dong *et al.* 2020).

## Conclusion

Genetic diversity exists among the 112 cowpea accessions evaluated in this study for drought tolerance and photosynthetic efficiency across environments. The strong, consistent correlation found between grain yield and some of the photosynthetic traits measured provides a valuable opportunity to introduce these traits in selection indices thereby harnessing the role of photosynthesis in genetic enhancement of cowpea tolerance to drought. This study showed the potential of GWAS in identifying MTAs for drought tolerance and photosynthetic efficiency in cowpea, and this provides an opportunity for the use of marker-assisted breeding methods to accelerate the development of drought-tolerant cowpea varieties and increase genetic gains in cowpea breeding programs. Furthermore, this study showed the importance of Gene Banks as a valuable resource for crop improvement and the application of genomics to unravel favorable alleles, which can support germplasm utilization by plant breeders in combating the increasing threat of climate change to sustainable food security.

## Data Availability

The phenotypic and genotypic dataset used in this study and the supplemental tables can be downloaded at figshare: https://doi.org/10.25387/g3.25550775. Supplementary Tables 1–3: List of cowpea accessions from the FIGS and minicore subsets including checks used for this study. Supplementary Table 4: Agronomic traits and mode of observation Supplementary Table 5: List of environments derived from water regime, location, and year. Supplementary Tables 6–8: Combined ANOVA tables for agronomic and photosynthetic traits measured across test conditions. Supplementary Tables 9–10: List of significant markers associated with agronomic and photosynthetic traits measured and their *P-values*. Supplemental material available at G3 online.
